# New Appliances and Things Medical

**Published:** 1894-01-20

**Authors:** 


### NEW APPLIANCES AND THINGS MEDICAL.

[All preparations, appliances, novelties, &c., of which a notice is desired, should be sent for the Editor, to care of The Manager, 428, Strand, London, W.C.]

### GERM WHITE BREAD.

We have received from the Malto-Germ Extract Company (Limited), 152, Bermondsey Street, London, S.E., a loaf and biscuits made from best flour with an admixture of "Rigola," or liquid extract of the germ of wheat. This extract contains the essentials of the germ or growing part of the corn, clevel, and not only has active properties of its own, but contains an extremely high percentage of phosphates, albumenoides, and fat. By its addition to roller-milled flour the bread receives that which the best modern flour is wanting in, and is correspondingly increased in nutritive value. The loaf and biscuits when they reached us were several days old, but there was a great absence of that dryness which ordinary bread of similar age would have. The flavour was excellent, quite bearing out the company's claim of being that of country farmhouse bread—we will add of the olden time, because much of the modern bread in farmhouses is made from modern milled flour, and so is absent in that excellent old-fashioned flavour with which we were familiar a good many years ago. We may add that the bread makes first-rate toast, another test of its goodness. Bad bread makes bad toast. The biscuits, both plain and sweet, were also of excellent quality. The company has certainly hit upon a most excellent invention, and we hope it will succeed.

